# Case Report: Contribution of [^18^F]FET PET in differential diagnosis between radionecrosis and progression in metastasis—reproducibility and superiority of dynamic acquisitions

**DOI:** 10.3389/fnume.2024.1287240

**Published:** 2024-02-29

**Authors:** Aurélien Callaud, Anne-Claire Dupont, Marie-Agnes By, Ilyess Zemmoura, Maria-Joao Santiago-Ribeiro

**Affiliations:** ^1^ Nuclear Medicine Department, CHRU de Tours, Tours, France; ^2^ UMR 1253, iBrain, Université de Tours, Inserm, Tours, France; ^3^ Oncology Department, CHRU de Tours, Tours, France; ^4^ Neurosurgery Department, CHRU de Tours, Tours, France

**Keywords:** [^18^F]FET PET, dynamic acquisition, cerebral metastasis, radionecrosis, progression

## Abstract

We present the case of a 67-year-old woman with metastatic invasive ductal carcinoma of the left breast, in whom a follow-up magnetic resonance imaging, 3 months after encephalic radiotherapy, revealed a significant increase in the size of two brain metastases, potentially indicating progressive disease within the radiation field. Subsequent [^18^F] fluorodeoxyglucose ([^18^F]FDG) and [^18^F] fluoroethyl-L-tyrosine positron emission tomography ([^18^F]FET PET) scans were performed to distinguish radionecrosis from tumor progression. Despite a dynamic [^18^F]FET time–activity curve (TAC) against progression, the exceeding of the 1.9 cutoff by mean tumor to brain ratio (TBR) and interdisciplinary considerations led to the resection of one lesion. Histopathology revealed necrosis due to radiotherapy, without viable tumor proliferation. To verify radionecrosis, a second [^18^F]FET PET scan was conducted, showing consistent findings. In metastasis differentiation, the mean TBR cutoff of 1.9 and TAC analysis achieved a sensitivity of 95% and specificity of 91%. The discrepancy between the TAC and TBR emphasizes the need for consideration, and a time delay between radiotherapy and PET may impact TBR cutoffs. In addition, differences in radiosensitivity suggest a lower metastasis pre-test probability of progression, and it might be why a TAC analysis could be more effective in distinguishing true progression from treatment related changes in metastasis. This case demonstrates the accuracy of dynamic [^18^F]FET PET and suggests its utility for post-treatment metastasis evaluation, and further research on post-treatment delay could lead to improved performances of dynamic [^18^F]FET PET.

We present the case of a 67-year-old woman with metastatic left breast infiltrating ductal carcinoma involving the bone and brain, with a 3-month post-radiotherapy (30–33 Gy in three fractions) follow-up magnetic resonance imaging (MRI) examination showing a significant increase in the size of two cerebral metastases (right frontal and left para-vermian superior cerebellar region) suggestive of potential progressive disease within the radiation field. It was difficult to definitively differentiate radionecrosis (RN) from tumor progression because of moderate neo-angiogenesis and extensive necrotic features. A subsequent [^18^F] fluorodeoxyglucose (FDG) positron emission tomography (PET), performed on a Vision 600 (Siemens Healthineers) with a 125 MBq injection, revealed only a large non-specific hypometabolic area in the right frontal region, superimposable to the edema on MRI (Figures 1, 2).

**Figure 1:**
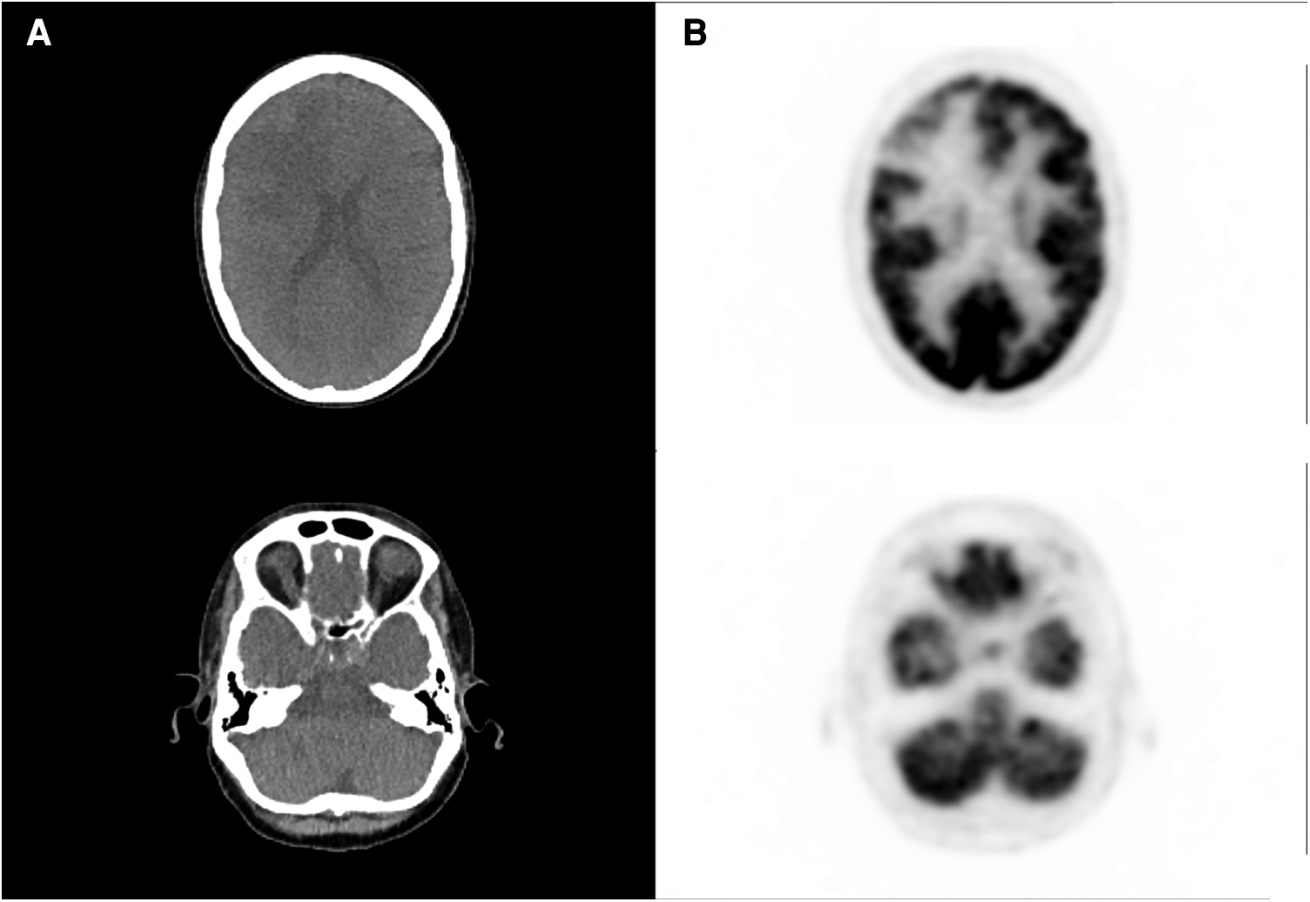
[^18^F]FDG PET and CT acquisition performed after the first doubtful MRI, showing large hypometabolic right frontal area (**A**) and no significant abnormal uptake in the left cerebellar region (**B**).

Therefore, a [^18^F] fluoroEthyl-L-tyrosine (FET) PET scan was performed on the same camera with a 147 MBq injection, showing a significant annular uptake of the right frontal region with a photopenic center and a mean tumor to brain ratio (TBR) of 2.03 (SUVmax = 2.7) and a significant uptake rounded left cerebellar lesion (SUVmax = 4.1) with a mean TBR of 2.48 (Figure 2A). Both values exceeded the reported mean TBR cutoff of 1.9, indicating potential tumor progression for frontal and cerebellum lesions (1), and time–activity curves (TACs) showing slow and progressive accumulating uptake (pattern I), with a time to peak (TTP) at 30 min or more, evocating this time radionecrosis (cerebellum and frontal before surgery TACs) (Figure 3) (1). However, given the pluridisciplinary information and the mean TBR remaining higher than the negative cutoff, it was decided to resect the frontal lesion (the cerebellar lesion was considered too deep and too small). The identification of lesion-related anatomical boundaries was performed using ultrasound, and dissection subsequently allowed for the localization of the lesion situated adjacent to the middle frontal gyrus. Macroscopically, it was rather suggestive of radionecrosis and was resected without complications. The final histopathological analysis revealed a necrotic lesion with inflammatory changes due to radiotherapy without viable tumor proliferation. To ensure that the cerebellum lesion was also radionecrotic despite the higher TBR and another follow-up MRI examination that was still doubtful, we performed a second [^18^F]FET PET scan 1 month postoperatively (Figure 2B), with a 144 MBq injection. In the surgical right frontal region (SUVmax = 3.4), a slightly higher mean TBR of 2.1 was found with an doubtful TAC (TTP at 20 min followed by a plateau, more suggestive of a pattern II than I) (Figure 3), in the context suggesting a non-specific accumulation probably inducted by postoperative large blood–brain barrier (BBB) rupture (postoperative frontal TAC) (Figure 3). Despite the progression of size on the last MRI scan, the cerebellar lesion (SUVmax = 5.0) had a similar mean TBR of 2.46 with a strictly identical curve, suggesting the absence of scalability (postoperative cerebellum TAC) (Figure 3). The most recent MRI scan showed a sagging of the right frontal excision cavity and discreet reduction of the flair hypersignal of the left cerebellar lesion rather in favor of radionecrosis, all still in agreement with the final conclusions from the [^18^F]FET PET scan (Figure 4).

**Figure 2:**
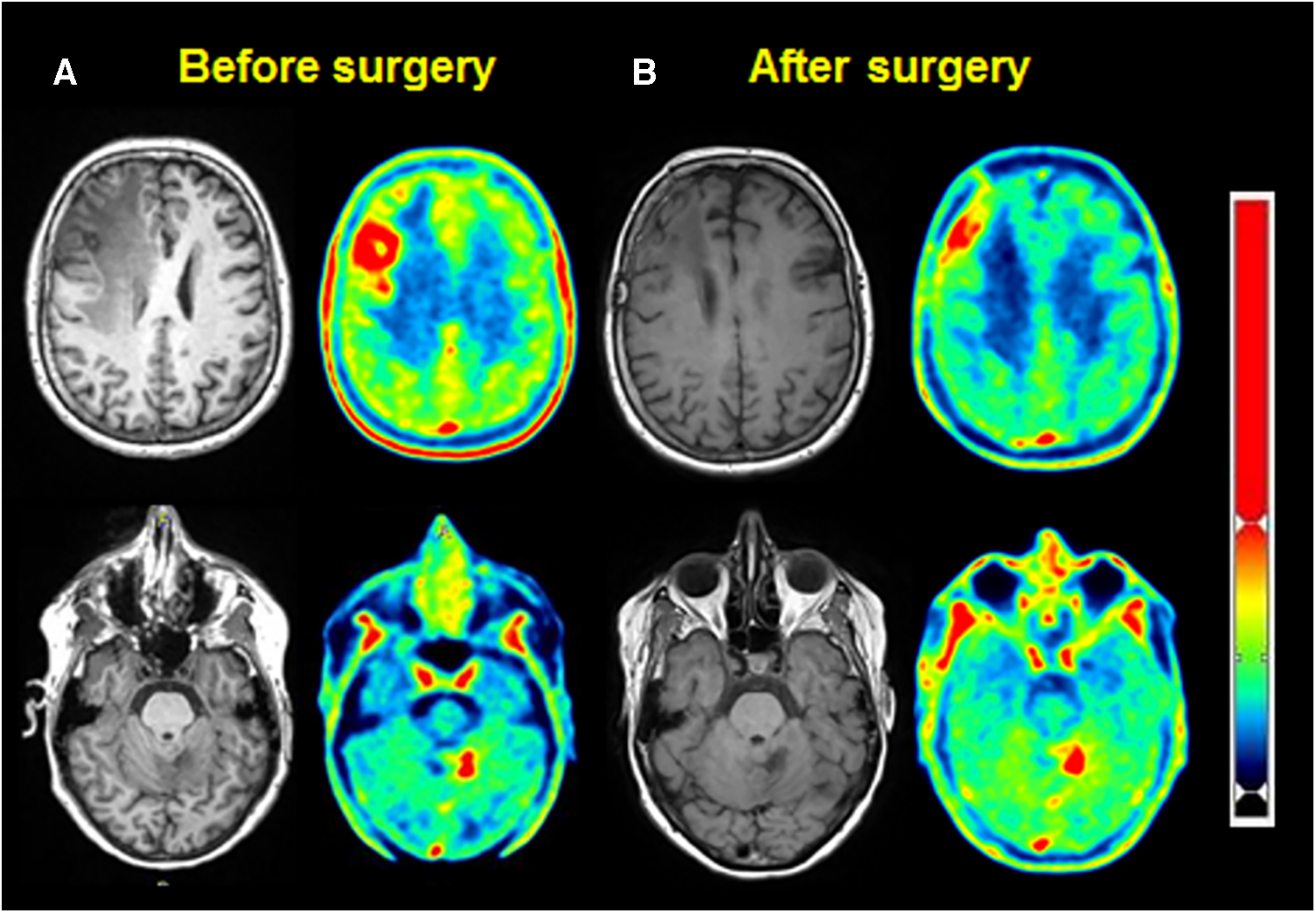
MRI (left) and [^18^F]FET PET (right), for frontal (up) and cerebellar (down) lesions, before (**A**) and after surgery (**B**).

**Figure 3:**
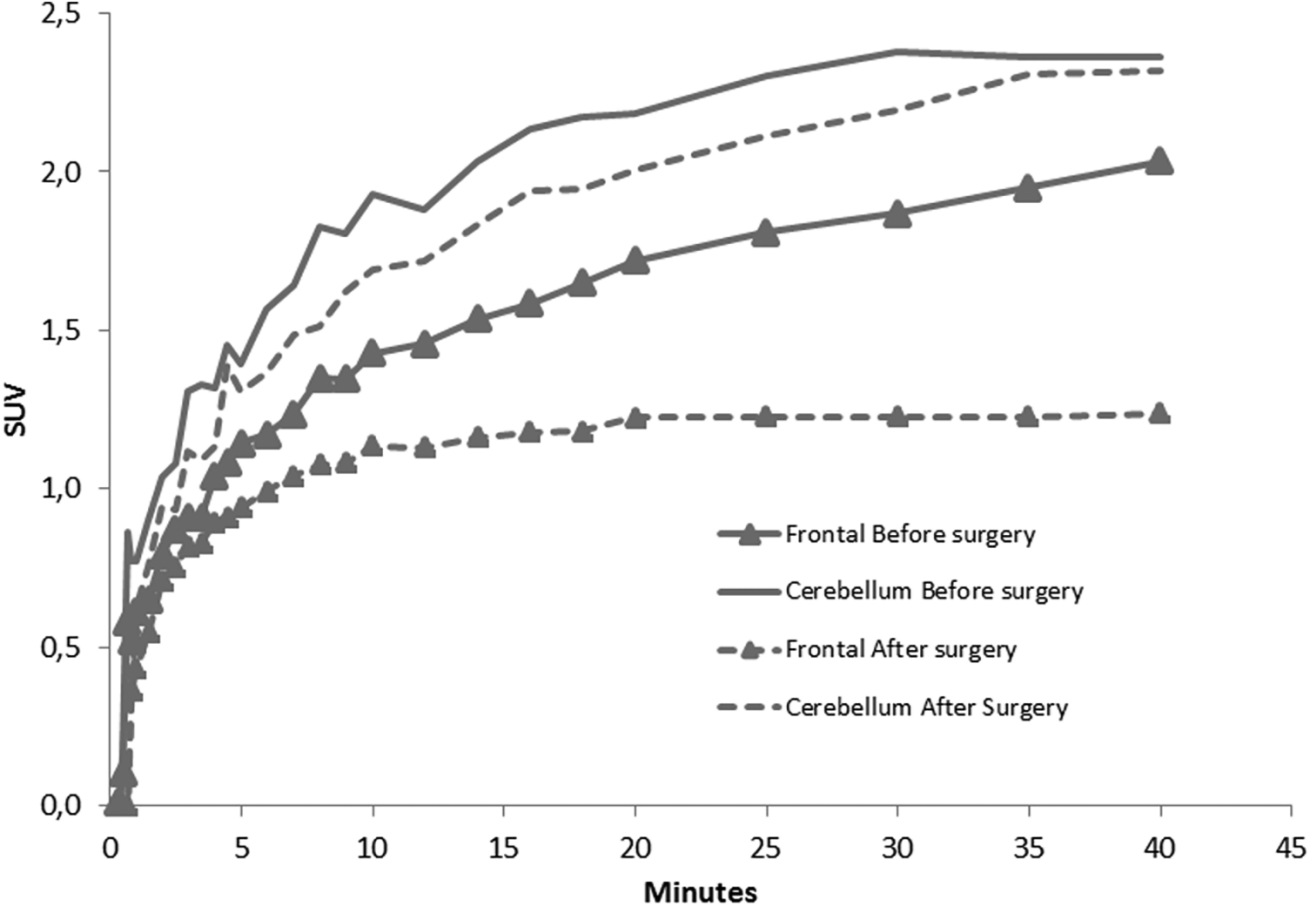
TACs of frontal and cerebellar lesion before and after surgery, showing the evolution of SUVmax during the first 40 min after [^18^F]FET injection.

**Figure 4:**
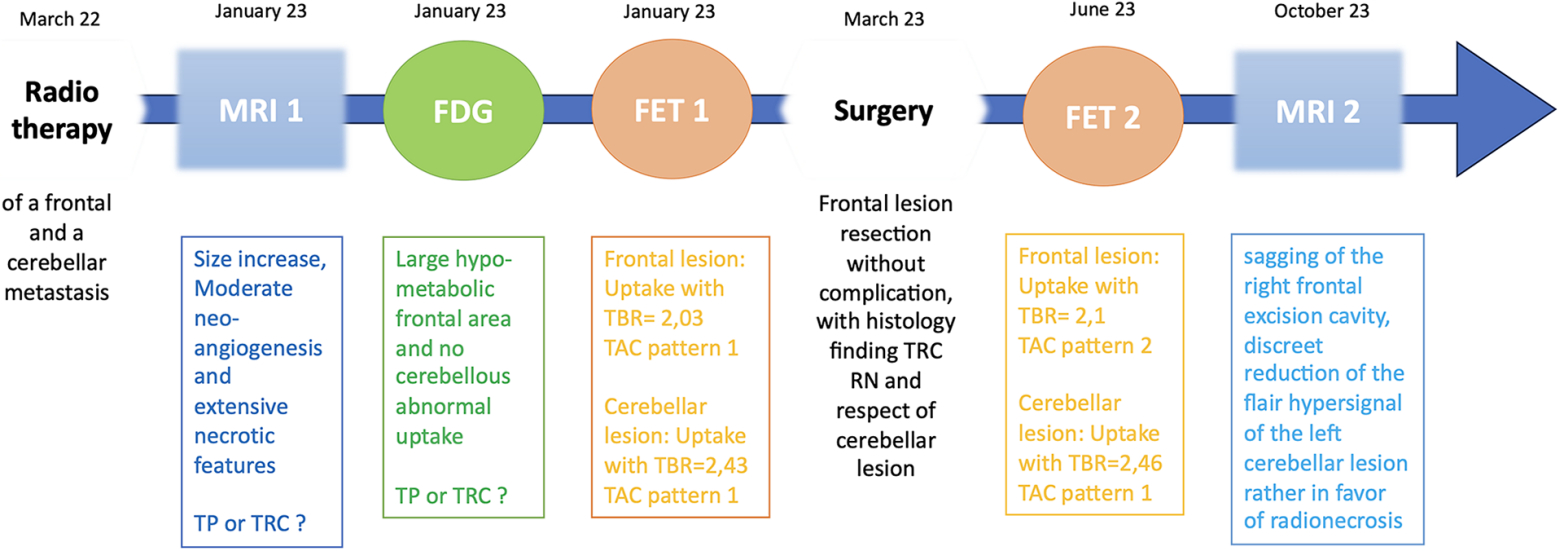
Timeline.

The analysis of TACs for the differentiation between true progression (TP) and treatment related changes (TRC) has already been studied (1), and it permitted the definition of three types of predictive TAC patterns. Pattern I corresponded to a slow progressive [^18^F]FET accumulation (with TTP > 20 min), pattern II corresponded to a faster accumulation followed by a plateau phase (TTP ≤ 20 min), and pattern III corresponded to a rapid accumulation followed followed by a progressive significant activity decrease (TTP ≤ 20 min). In the context of metastases, the best performances for the distinction of TP from TRC were reported previously by combining both a mean TBR cutoff of 1.9 and an analysis of TAC (pattern I vs. II or III), enabling a sensitivity of 95% and a specificity of 91% (1). Here we have a discordance between TAC and mean TBR, highlighting two points. First, to distinguish TP and TRC in glial tumors, European guidelines report different mean TBR cutoffs based on early or late post-treatment evaluation (±3 months from treatment), with respective cutoffs at 2.3 and 1.9 (2–4). In the study by Galldiks et al. (1), the median time between treatment and PET scan was 11.5 months, which is later than in our study, and could be one of the hypotheses for the existence of a higher mean TBR in our case. Like the early and late distinction for gliomas, considering the time delay between radiotherapy and [^18^F]FET PET could be relevant for TBR cutoffs related to metastases.

Furthermore, performing a TAC analysis appears to be equally if not more discriminating than TBR for distinguishing TP and TRC (1, 2). The TAC analysis seems to be unaffected by the glial or metastatic tumor nature, since the distinction of TP and TRC is based on the same patterns, because the explored transporters are identical [L-type amino acid transporter (LAT), sodium-coupled neutral amino acid transporters (SNAT), alanine serine cysteine transporters (ASCT)]. We can also emphasize here that the excellent reproducibility between the two TACs of the cerebellar lesion separated by several months, reflecting the reliability of amino acid transporter imaging, allows the overcoming of many contextual factors that could be problematic with [^18^F]FDG, for example. Despite better overall performances, this analysis also has its limitations, as shown here by the immediate postoperative curve of the frontal lesion, closer to pattern II with a TTP of 20 min, which, according to previously reported data, would suggest a recurrence instead (1).

Another important consideration is the generally higher radiosensitivity of metastasis compared to primary glial tumors, with a higher dose by fraction delivered to metastasis, and the less infiltrative character of metastasis, resulting in better response rates and a higher probability of developing radionecrosis or pseudoprogression features with metastasis treatment (5). Therefore, the suspicion of metastasis progression might be evaluated considering this lower pre-test probability. It might be another reason why dynamic [^18^F]FET PET acquisition could be more relevant for metastasis.

This example clearly illustrates the accuracy of dynamic [^18^F]FET PET performances, which should justify its implementation in clinical routines. Conducting additional investigations into the post-treatment delay preceding [^18^F]FET PET may serve as a valuable avenue for improving the performances of dynamic [^18^F]FET PET in the post-treatment evaluation of metastasis.

## Data Availability

The original contributions presented in the study are included in the article/Supplementary Material, further inquiries can be directed to the corresponding author.
